# A roadmap for better and personalized mental health care in Europe: the priorities of the European Psychiatric Association

**DOI:** 10.1192/j.eurpsy.2025.2456

**Published:** 2025-05-23

**Authors:** Andrea Fiorillo

**Affiliations:** President, European Psychiatric Association (EPA), Department of Psychiatry, University of Campania “Luigi Vanvitelli”, Naples, Italy

In the last few years, European citizens have been facing several social and political challenges, such as the widespread outbreak of the COVID-19 pandemic, the breakout of conflicts at European borders, the consequences of the economic recession, and the forced migrations of people from countries stricken by wars and poverty, which have all had documented detrimental effects on mental health. These challenging situations have added a significant burden to the mental health of the general population, particularly to individuals already suffering from mental disorders. According to recent research [1], since 2021, the levels of anxiety, depression, and loneliness have risen by about 25%, with a consequent increase in personal, social, and family burden. Moreover, a 30.9% increase in years lived with disability was reported between 2019 and 2021 in all European countries, with direct and indirect costs for mental disorders accounting for 4.1% of the gross domestic product of European Union Member States and the United Kingdom. According to the 2018 report of the *Lancet* Commission on global mental health and sustainable development [2], high- and low-income countries should allocate 10% and 5% of their respective healthcare budget to mental health care. However, this target is far from being achieved by most European countries, resulting in a gap of EUR 25.9 billion.

Serious consequences of this increased prevalence of mental disorders in Europe include a higher rate of mortality due to mental and behavioral disorders (~235,000 deaths per year) and suicide (~140,000 cases of suicide per year), which represents the fourth leading cause of death among people aged 20 years or younger [1].

Under these circumstances, a call for immediate action is needed, with practical strategies to be implemented to promote well-being and improve mental health for all European citizens. Concrete actions include increased investment in human resources and infrastructure, the development of new models and settings of care, the delivery of mental health interventions in different settings (e.g., at school or workplace) or through the use of new technologies (e.g., virtual reality or digital tools), and the promotion of awareness-raising campaigns to reduce the impact of psychosocial risk factors on vulnerable groups (e.g., children and adolescents, migrants, and discriminated groups).

In December 2023, the European Psychiatric Association (EPA) launched a Manifesto highlighting the importance of promoting and harmonizing mental health care standards across Europe [3]. The Manifesto, which was subsequently endorsed by the European associations of experts-by-experience (GAMIAN-Europe) and of family members and caregivers (EUFAMI), identified five key areas for immediate action:

1. to harmonize mental health care delivery;
2. to improve working conditions and address shortages in the mental health workforce;
3. to promote and harmonize ethical standards;
4. to develop new answers to an evolving world;
5. to promote research and implementation of public mental health and prevention measures [4].

Building on these foundations, the new EPA Action Plan 2025–2027 “Leaving no one behind – a roadmap for better and personalized mental health care” will address six areas, briefly reported below.

1. *Treatment delivery and new settings of care.* One out of four European citizens reports that they or their family members face barriers to accessing mental health care, such as long waiting lists and high treatment costs [5]. The shortage of mental health professionals represents an issue in most European countries. Many infrastructures are outdated and fail to meet the needs of young people suffering from mental health problems. There is a need for innovative and stigma-free models of caring, including the use of new technologies and services that are easily accessible to everyone [6]. Psychiatrists should consider providing mental health care in non-traditional settings, including schools, workplaces, and jails [7–9].
2. *Precision psychiatry.* For more than a century, psychiatric diagnoses have usually been made in the presence of signs and symptoms for a certain period, which cause some (degree of) distress. We are still far from the identification of clear biomarkers or brain dysfunctions in people affected by any mental disorder. Therefore, we should aim at identifying those genetic, biological, or psychosocial stressors that can alter physiological brain development and aging – from prenatal life to childhood and adolescence, and from adulthood to the elderly [10, 11]. A full assessment of several domains (e.g., neurocognition, physical comorbidities, presence of major life events, or illness staging) might represent a first step toward more precise psychiatric diagnoses [12]. Furthermore, the development of new drugs targeting pathophysiological mechanisms (such as melatonin, orexin, GABA-A, opioid and N-methyl-D-aspartate receptor systems, the vesicular monoamine transporter2, and inverse agonism of 5HT2A receptors) rather than enhancing serotonin and noradrenaline or blocking postsynaptic dopamine transmission for the treatment of mental disorders promises, in the near future, the availability of pharmacological treatments adapted to different patients’ biotypes [13]. The identification and use of biomarkers in clinical practice will hopefully lead to the early identification of specific disease profiles and stages, thus optimizing tailored individual treatments [14], and modifying our diagnostic and therapeutic approaches.
3. *Brain and mental health across the lifespan.* All mental disorders stem from an altered organ (i.e., the brain); therefore, the study of brain functioning (and of brain dysfunctions) should be encouraged. We cannot overlook the pathophysiology of the developing brain, particularly in youth and adolescence, when most mental disorders have their onset. Brain activities typically occurring in early adolescence, such as myelinization and pruning, should be considered when initiating psychiatric treatments. Research on brain health should be fostered, given the similarities between several mental disorders (e.g., schizophrenia) and several neurological diseases (e.g., multiple sclerosis) [15], and the impact of some physiological brain activities, such as sleep, on mental health and well-being [16].
4. *Lifestyle behaviors, physical and mental health, and multimorbidity.* People with severe mental disorders have poor physical health and a reduced life expectancy of 15–20 years compared with the general population, a gap that has been described as a public health scandal [17]. This mortality gap is due to several factors, including individual ones, such as lifestyle behaviors and social disadvantages, and systemic ones, such as reduced access to healthcare facilities and stigma among health professionals [18]. Focusing on the physical health of people with severe mental disorders means improving training and education on the physical health domains, fostering collaboration with other physicians’ associations, and implementing, at the European level, the so-called “lifestyle psychiatry”, including psychosocial interventions aiming to improve patients’ lifestyle [19].
5. *Public mental health.* This working group will focus on the prevention of mental disorders, the promotion of mental health in the general population, scalable interventions for people living in communities affected by adversities, and fighting stigma and discrimination. Since 74% of mental disorders occur by the age of 24, the working group will focus on adolescents and young adults. Mental health interventions should be more intense in youth during the “critical period” [20], when the illness develops and the disability plateaus, with serious long-term consequences if proper treatment is not delivered. Investing in youth mental health is a “best buy” requiring stronger attention from clinicians, researchers, educators, and policymakers [21]. Promotion of mental health also involves driving social and cultural changes, made possible through initiatives aimed at fighting stigma and disseminating knowledge and self-awareness. Mass media and social networks can support this by adopting an effective, accurate, and stigma-free language, as well as by giving voices to mental health professionals and experts by experience.
6. *Mental health protection of vulnerable groups.* The risk of developing mental health problems is higher in marginalized or discriminated people, such as migrant or displaced populations, LGBTQI+ persons, women (in some countries), and other disadvantaged groups. In particular, migrants are heavily exposed to psychotic, trauma-related, and affective disorders due to several stressors in the pre-, peri-, and post-migratory phases [22]. More than 50% of LGBTQI+ persons report a lifetime episode of major depression or anxiety disorders [23]. Those living in conflict-affected countries report significantly higher levels of burden, distress, and mental disorders. Thus, there is a need to provide tailored, evidence-based interventions to these groups and to create a stigma-free culture all over Europe.

The six tasks of the EPA Action Plan will be addressed through the involvement of European experts (including users and carers), who will work at three different levels: education, clinical practice, and advocacy. Each task force will produce consensus documents, statements, clinical guidelines, and educational materials, as well as surveys and research projects, with the involvement of some of the most important European scientific associations and advocacy groups. The ultimate goal of the EPA is to participate in and contribute to a cultural, political, and societal change aimed at improving the mental health and well-being of everyone living in Europe.
